# Myocarditis and Other Cardiovascular Complications of the mRNA-Based COVID-19 Vaccines

**DOI:** 10.7759/cureus.15576

**Published:** 2021-06-10

**Authors:** Mahesh K Vidula, Marietta Ambrose, Helene Glassberg, Neel Chokshi, Tiffany Chen, Victor A Ferrari, Yuchi Han

**Affiliations:** 1 Medicine/Cardiology, Hospital of the University of Pennsylvania, Philadelphia, USA

**Keywords:** covid-19 disease, myocarditis, stress cardiomyopathy, pericarditis, vaccine

## Abstract

Cardiovascular complications following the receipt of mRNA-based (Pfizer-BioNTech and Moderna) coronavirus disease 2019 (COVID-19) vaccines have not yet been described. In this case series, we describe two patients with clinically suspected myocarditis, one patient with stress cardiomyopathy, and two patients with pericarditis after receiving an mRNA-based COVID-19 vaccine. The two patients with clinically suspected myocarditis were otherwise healthy young men who presented with acute substernal chest pressure and/or dyspnea after receiving the second dose of the vaccine and were found to have diffuse ST elevations on electrocardiogram (ECG), elevated cardiac biomarkers and inflammatory markers, and mildly reduced left ventricular (LV) function on echocardiography. Both patients met the modified Lake Louise Criteria for acute myocarditis by cardiac magnetic resonance imaging. We subsequently discuss a case of a 60-year-old woman with known coronary artery disease (CAD) and previously normal LV function, who presented with new exertional symptoms, ECG changes, and apical akinesis following the second dose of the vaccine, and was diagnosed with a stress cardiomyopathy. Finally, we describe two patients with pericarditis who presented with chest pain, elevated inflammatory markers, and pericardial effusions after receiving the vaccine. Overall, this case series describes the first reported cases of myocarditis, stress cardiomyopathy, and pericarditis after receiving an mRNA-based COVID-19 vaccine.

## Introduction

In December 2020, the Food and Drug Administration issued an Emergency Use Authorization (EUA) for two mRNA-based vaccines developed by Pfizer-BioNTech and Moderna for the prevention of coronavirus disease 2019 (COVID-19) [1]. In the clinical trials assessing the safety of these two-dose vaccines, patients who received the vaccines more commonly experienced higher rates of local injection site reactions, fatigue, and headaches, and there were very few serious adverse events [2,3]. Furthermore, there were no reports of serious cardiovascular events. 

Although prior studies have shown that cardiovascular complications, such as myocardial infarction, myocarditis, ventricular arrhythmias, and stress cardiomyopathy, were prevalent in hospitalized patients with COVID-19 [4-6], cardiovascular complications of the mRNA-based vaccines have not yet been reported in the literature, and are likely to be very rare. In this case series, we describe two patients with clinically suspected myocarditis, one patient with stress cardiomyopathy, and two patients with pericarditis after receiving an mRNA-based COVID-19 vaccine. None of these patients had previously tested positive for severe acute respiratory syndrome coronavirus 2 (SARS-CoV-2).

## Case presentation

Myocarditis

Case 1

A 19-year-old male student with no significant past medical history presented to our hospital with acute substernal chest pressure and shortness of breath four days after receiving the second dose of the Pfizer-BioNTech vaccine.

**Figure 1:**
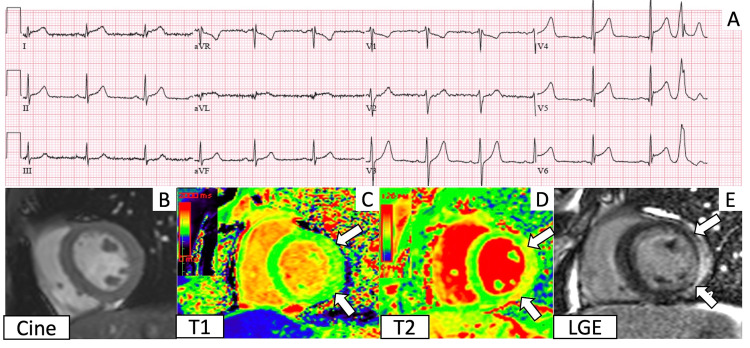
Electrocardiogram (ECG) and cardiac magnetic resonance imaging (CMR) findings corresponding to Case 1 A. ECG shows diffuse ST elevations. B. Short axis cine in diastole. C. T1 imaging demonstrates regional elevation in the lateral wall (between the arrows). D. T2 mapping shows elevated T2 values in the lateral wall (between the arrows). E. Late gadolinium enhancement imaging shows subepicardial enhancement (between the arrows) in the same areas of T1 and T2 elevations.

On presentation, he was afebrile, with a heart rate of 71 beats per minute (bpm), and blood pressure of 145/95 mmHg. Physical exam was unremarkable. His electrocardiogram (ECG) was notable for diffuse ST elevations (Figure 1A), and laboratory studies demonstrated evidence of myocardial injury and elevated inflammatory markers (Table 1). There was no evidence of coronary artery disease (CAD) on coronary angiography, and echocardiography showed a mildly reduced left ventricular (LV) ejection fraction (EF) of 47% with no regional wall motion abnormalities.

**Table 1:** Summary of diagnostic testing. CRP, C-reactive protein; ESR, erythrocyte sedimentation rate; HIV, human immunodeficiency virus; LAD, left anterior descending artery; LGE, late gadolinium enhancement; LVEDVi, left ventricular end-diastolic volume index; LVEF: left ventricular ejection fraction; LVIDd: left ventricular end-diastolic internal dimension; MRI, magnetic resonance imaging; RSV, respiratory syncytial virus; RVEDVi, right ventricular end-diastolic volume index; RVEF: right ventricular ejection fraction

Cardiovascular Adverse Event	Age (years)	Gender	Viral serologies	Cardiac Biomarkers	Inflammatory Markers	Transthoracic Echocardiogram	Cardiac MRI	Coronary Evaluation
Myocarditis								
Patient 1	19	Male	HIV, influenza, RSV, and SARS-CoV-2 negative	Troponin T: 1.37 ng/mL (reference range: <0.03 ng/mL)	CRP: 1.8 mg/dL (reference range: <0.80 mg/dL); ESR: 26 mm/hour (reference range: < 20 mm/hour)	LVEF: 47%; LVIDd: 4.7 cm; Normal RV size and function; No valvular disease	LVEF: 56%; LVEDVi: 84 mL/m^2^; RVEF: 54%; RVEDVi: 86 mL/m^2^; Subepicardial LGE involving the basal to mid lateral wall, with corresponding elevated native T1 and T2 values	Coronary angiography: No coronary artery disease or anomalies
Patient 2	18	Male	HIV, influenza, RSV, and SARS-CoV-2 negative	High-sensitivity troponin: 7,206 ng/L $\rightarrow$ 32,140 ng/L (reference range: <15 ng/L)	CRP: 74.2 mg/L (reference range: <10 mg/L); ESR: 29 mm/hour (reference range: <15 mm/hour)	LVEF: 59% $\rightarrow$ 50%, LVIDd: 4.8 cm; Normal RV size and function; No valvular disease	LVEF: 53%; LVEDVi: 101 mL/m^2^; RVEF: 49%; RVEDVi: 118 mL/m^2^; Subepicardial LGE involving the mid lateral wall, with corresponding elevated native T1 and T2 values	Coronary computed tomography angiography: No coronary artery disease or anomalies
Stress Cardiomyopathy								
Patient 1	60	Female	SARS-CoV-2, influenza, and RSV negative	Troponin T: 0.129 ng/mL (reference range: <0.03 ng/mL)	Not checked	LVEF: 44%; LVIDd: 3.5 cm; Normal RV size and function; Apical akinesis	Not performed	Coronary angiography: Patent LAD stent
Pericarditis								
Patient 1	21	Female	SARS-CoV-2, HIV negative	Troponin T: undetectable	CRP: 72.6 mg/L (reference range: <3 mg/L); ESR: 9 mm/hour (reference range: <19 mm/hour)	LVEF: 60%; LVIDd: 4.5 cm; Normal RV size and function; Small pericardial effusion	Not performed	Not performed
Patient 2	61	Female	SARS-CoV-2 negative	Troponin T: undetectable	CRP: 23.1 mg/dL (reference range: <0.80 mg/dL); ESR: 81 mm/hour (reference range: <15 mm/hour)	LVEF: 65%; LVIDd: 3.6 cm; Normal RV size and function; Small to moderate circumferential pericardial effusion	Not performed	Not performed

Cardiac magnetic resonance imaging (CMR) identified mild hypokinesis of the basal to mid lateral wall, with corresponding elevated native T1 values (1070-1160 ms; reference range: 950-1050 ms), elevated T2 values (57-59 ms; reference range: 40-50 ms), and subepicardial delayed enhancement (Figure 1B-1E) in the lateral wall. Based on the clinical presentation and updated Lake Louise Criteria for myocardial inflammation by CMR [7], the patient was diagnosed with acute myocarditis. Subsequent viral studies were negative for human immunodeficiency virus (HIV), influenza, respiratory syncytial virus (RSV), and SARS-CoV-2. The patient’s chest pain resolved, and he was discharged on lisinopril and metoprolol succinate.

Case 2

An 18-year-old male student with no significant past medical history was transferred to our hospital for management of worsening LV function following the second dose of the Moderna vaccine.

**Figure 2:**
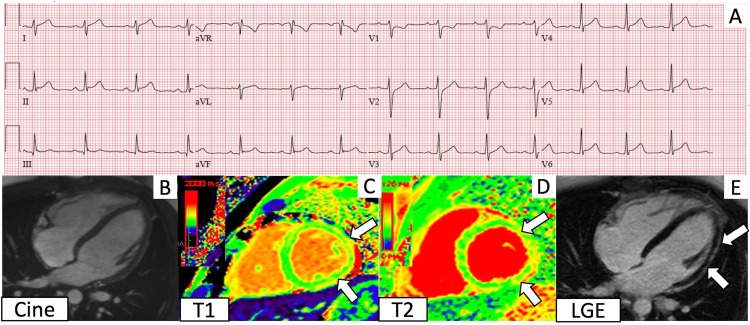
Electrocardiogram (ECG) and cardiac magnetic resonance imaging (CMR) findings corresponding to Case 2 A. ECG shows diffuse ST elevations. B. 4 chamber cine. C. T1 imaging demonstrates regional elevation in the lateral wall (between the arrows). D. T2 mapping shows elevated T2 values in the lateral wall (between the arrows). E. Late gadolinium enhancement imaging shows subepicardial enhancement (between the arrows) in the same areas of T1 and T2 elevations.

He initially presented to another hospital, where he reported fevers, myalgias, and acute substernal chest pain one day after receiving the second dose. On presentation, his temperature was 101°F, heart rate was 120 bpm, and blood pressure was 110/59 mmHg. Physical examination was unremarkable. ECGs demonstrated diffuse ST elevations (Figure 2A), and laboratory studies were notable for an elevated high-sensitivity troponin. LV systolic function on the initial echocardiogram was normal, but a subsequent echocardiogram demonstrated a mild reduction in LVEF to 50%. At our hospital, coronary computed tomography angiography revealed normal coronaries.

CMR revealed mild hypokinesis of the mid lateral wall, with corresponding elevated native T1 values (1089-1097 ms; reference range: 950-1050 ms) and T2 values (64-72 ms; reference range: 40-50 ms), and subepicardial delayed enhancement (Figure 2B-2E) in the lateral wall. Viral serologies for HIV, influenza, RSV, and SARS-CoV-2 were negative. The patient’s hospital course was complicated by brief episodes of non-sustained ventricular tachycardia, and he was discharged on metoprolol succinate and a course of colchicine and ibuprofen. 

Stress cardiomyopathy

**Figure 3:**
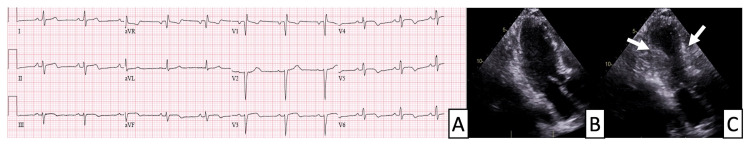
Electrocardiogram (ECG) and echocardiography findings in a patient with stress cardiomyopathy A. ECG demonstrating T wave abnormalities in the inferolateral leads (II, III, aVF, V3-V6). B. Apical 3 chamber view in diastole. C. Apical 3 chamber view in systole. The arrows denote the hinge points of apical akinesis.

A 60-year-old woman with a history of a stent placed in the left anterior descending artery (LAD) three years ago, and normal LV function and wall motion on echocardiography five months ago, presented to clinic four days after receiving her second dose of the Pfizer-BioNTech vaccine, with exertional chest pain and new inferolateral T wave inversions (Figure 3A). She was admitted to the hospital for further evaluation, and echocardiography revealed mildly reduced LV function with apical akinesis (Figure 3B-3C). Coronary angiography demonstrated a patent LAD stent and no obstructive disease. Based on these findings, the patient was diagnosed with a stress cardiomyopathy, and she was discharged on metoprolol succinate and lisinopril.

Pericarditis

The first patient was a 21-year-old woman with a history of idiopathic thrombocytopenic purpura, who presented three weeks after her first dose of the Pfizer-BioNTech vaccine with chest pain that worsened during inspiration and while supine. She was found to have sinus tachycardia, a small pericardial effusion on echocardiogram, and elevated inflammatory markers without evidence of myocardial injury. Based on the clinical presentation, the patient was diagnosed with pericarditis [8]. Her symptoms improved significantly with initiation of colchicine. 

**Figure 4:**
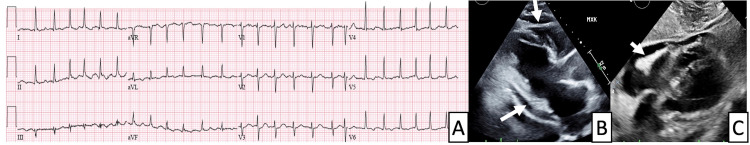
Electrocardiogram (ECG) and echocardiography findings in a patient with pericarditis A. ECG demonstrates atrial fibrillation and nonspecific T wave changes. B and C are the parasternal long axis view and subcostal view, respectively. Arrows point to the circumferential pericardial effusion.

The second patient was a 61-year-old woman with a history of hypertension who developed low-grade fevers, night sweats, chest discomfort and palpitations four weeks after her second dose of the Pfizer-BioNTech vaccine. She noted that her symptoms improved when leaning forward. On exam, she was found to have a friction rub. ECG revealed new-onset atrial fibrillation (Figure 4A) and echocardiography demonstrated a small to moderate circumferential pericardial effusion (Figure 4B-4C). Inflammatory markers were significantly elevated and viral serologies were negative. Based on these clinical features, the patient was diagnosed with pericarditis [8]. The patient was started on colchicine with improvement in her symptoms. Both patients tested negative for SARS-CoV-2.

## Discussion

In this case series, we describe patients with clinically suspected myocarditis, stress cardiomyopathy, and pericarditis after receiving an mRNA-based COVID-19 vaccine. Since December 2020, over 865,000 residents in our metro area have been vaccinated [9]. The findings we discuss in this report are rare adverse cardiovascular events, which were not previously identified or reported in the large safety and efficacy trials. In addition, among 127,496 adverse events reported to the Vaccine Adverse Event Reporting System (VAERS) between December 2020 and May 2021, there were 552 (0.4%) reported cardiovascular events and 103 were labeled as myocarditis, 15 as stress cardiomyopathy and 106 as pericarditis. However, the VAERS data is limited by volunteer reporting and lacks adjudication, and therefore the true incidences of these events in the vaccinated population are not known but are likely exceedingly low [10].

Since December 2019, SARS-CoV-2 has infected over 167 million people worldwide [11]. Cardiac injury, including myocardial infarction, myocarditis, and stress cardiomyopathy, was prevalent in hospitalized patients and portended a worse prognosis [4-6]. Myocarditis is defined as myocardial inflammation with histological evidence of inflammatory infiltrates and evidence of necrosis, and may be the result of a variety of etiologies including infections and toxins [12]. While endomyocardial biopsy was not performed in the two patients discussed above, both patients met the European Society of Cardiology criteria for clinically suspected myocarditis and the CMR updated Lake Louise Criteria, which increased the specificity for the diagnosis of myocarditis [7].

While the definitive etiologies for these cases cannot be identified, the temporal association of the receipt of the vaccine and absence of other plausible causes suggest the vaccine as the likely precipitant of these rare events. Myocarditis and pericarditis are rare cardiovascular complications that have also been associated with vaccines such as the smallpox and influenza vaccines, and it has been postulated that the systemic inflammatory response to immunizations can lead to myocardial and pericardial inflammation [13,14]. There have been reports of immune activation from influenza vaccination leading to stress cardiomyopathy as well [15]. Long-term follow-up of patients with cardiovascular events following the receipt of mRNA-based COVID-19 vaccines is needed. At the time of publication, all patients presented in this case series demonstrated clinical improvement.

## Conclusions

We report the first case series of patients with myocarditis, stress cardiomyopathy, and pericarditis after receiving the mRNA-based COVID-19 vaccines. While we cannot definitively prove association of the vaccines with these adverse events, we believe reporting these complications may enable further monitoring and investigation. However, it is crucial to emphasize that these findings are rare while SARS-CoV-2 infection is associated with a much higher incidence of cardiovascular complications.
